# Toward Molecular Level of the “*Salmonella*-Victim” Ecology, Genetics, and Evolution

**DOI:** 10.1100/tsw.2004.20

**Published:** 2004-03-15

**Authors:** S.N. Rumyantsev

**Affiliations:** Andent, Inc., Waukegan, IL, 60085, USA

**Keywords:** constitutional immunity, constitutional sensitivity, cytolysin, genetic diversity, genetic epidemiology, human twins, infection, microbe-victim ecology, microbe-victim evolution, microbial pathogenesis, molecular ecology, Salmonella

## Abstract

Bacteria of the *Salmonella* genus are polypathogenic agents that can affect both men and animals, causing devastating and fatal illness. Despite considerable immunological, epidemiological, and genetic efforts, and increased understanding of how the *Salmonella* infection develops, many key questions concerning *Salmonella* infection remain unanswered. *Salmonella* can be carried as harmless commensals in some sectors of the population. In some individuals, however, the same microbes cause illness while others display immunity to primary *Salmonella* infection. Nothing is known about the molecular base of the *Salmonella* pathogenicity. Even the ability of *Salmonella* to destroy the victims cells has been the subject of century-long discussions. In this article, some key findings concerning ecology, molecular ecology, and cell level of the *Salmonella* infection genetics are summarized and interpreted from the viewpoint of evolutionary theory with certitude that this approach can help to decipher the undiscovered secrets of *Salmonella* infections epidemiology and pathogenesis, as well as the clinical course and severity, and to select ways for fighting against *Salmonella*.

## TODAY'S SIGNIFICANCE AND ISSUES OF SALMONELLOSES

Bacteria of the *Salmonella* genus are polypathogenic agents that can affect both men and animals, causing significant morbidity and mortality. According to a WHO estimate, annually there are more than 17,000,000 *Salmonella* infection cases, associated with about 600,000 deaths[1]. Like any infectious agent, *Salmonella* cannot cause illness in all members of an observed species or population. *Salmonella* can be carried as harmless commensals in some sectors of the population. In some individuals, however, the same microbes cause devastating and fatal illness. Volunteer challenge studies have established a minimal infective dose for *S. typhi* of 100,000 to 1,000,000,000 microbial cells[2]. Reported mortality associated with *Salmonella* infection varies between different ethnic populations. For instance, the incidence of mortality due to *S. typhi* infection in Indonesia and New Guinea is higher than in other countries in southeast Asia[3,4], although all *S. typhi* isolates from different areas of the world can be classified as clones that have spread from a single source[5]. Some organisms attacked by *Salmonella* fall ill, whereas many individuals, families, strains, races, populations, and species display immunity to primary *Salmonella* infection.

Despite considerable immunological, epidemiological, and genetic efforts, and increased understanding of how the *Salmonella* infection develops in the clinical course, many key questions concerning *Salmonella* infection remain unanswered. The disease continues to be a life-threatening emergency. Nothing is known about the molecular base of the *Salmonella* pathogenicity. Even the ability of *Salmonella* to destroy the victim's cells has been the subject of century-long discussions. Accordingly, there have been no approaches toward discovering the molecular origin of the species and individual differences in susceptibility to primary *Salmonella* infection. The recent unlocking of the genome of the human typhoid bacillus[5] did not add anything new to the indigent knowledge of *Salmonella* infection molecular pathogenesis or natural susceptibility. At the same time, there is a limited body of research focusing on the relevant genetic features of the victims of *Salmonella* infection.

In this article, the key findings concerning ecology, molecular ecology, and cell level of *Salmonella* infection genetics are summarized and interpreted from the viewpoint of evolutionary theory with certitude that this approach can help to decipher the undiscovered secrets of *Salmonella* infection's epidemiology and pathogenesis, as well as clinical course and severity.

## GENERAL AND MOLECULAR ECOLOGY OF THE "*SALMONELLA*-VICTIM" SYSTEM

Unlike genetics of pathogenic microbes, the topic of infectious diseases ecology, genetics, and evolution has been presented still recently by only a few monographic publications. This situation has now begun to change. After being neglected for a long time, the principles of molecular ecology and evolution have now become a quickly re-emerging branch of scientific investigation of "microbe-victim" ecological systems. The special journal entitled *Infection, Genetics and Evolution* began to be published[6]. Principles of ecology and evolution began to function as effective tools of discovery and interpretation.

The harmful micro-organisms usually penetrate the animal or human victims using one of the main channels of its interactions with the environment (nutritional, respiratory, or sexual). The type of this ecological interface is strongly predetermined by previous evolutionary coadaptation of both microbe and victim, and is characteristic of every specific kind of "microbe-victim" ecological system. In the case of the "*Salmonella*-victim" ecological system, the infectious agents penetrate a victim's body by means of the nutritional channel of its ecological connections. After infection develops, the victim's body becomes the source of *Salmonella* multiplication and further transmission. The diseased victim and its corpse become the source of the next circle of *Salmonella* transmission in the body of a next victim. Thus the aptitude for regular interaction with this kind of infectious agent is characteristic of omnivores and carnivores instead of herbivores[7]. This postulate is very important for discovery of the molecular level of the "*Salmonella*-victim" ecological system.

Although it was supposed that like many other intracellular pathogens, the representatives of the *Salmonella* genus "have an inherent capacity to breach host cell barriers"[8] and display a wide range of virulence, nothing of the factors that enable the bacteria to infect and damage the victims' structures and functions have been revealed until recently. Some investigators have occasionally observed, for instance, the cytolytic capacity of certain *Salmonella* strains in agar plates containing sheep or human blood[9,10,11,12], but these observations were not confirmed by other researchers[13,14] although both groups of investigators made use of the same object, namely sheep blood. A *Salmonella* gene encoding a specific toxin (cytolysin) was once identified by screening for hemolysis on sheep blood agar[10], but this finding was soon discredited[13,15]. The *Salmonella* pathogenetic ecological agents remain unknown for the world scientific community, as well as the phenotypic determinants of the genetic diversity in specific and individual predisposition to *Salmonella* infection and the factors by which it is conditioned. Up to 2002[15], this topic remained a very poorly understood area of the pathogenesis, molecular ecology, and unity in salmonelloses. Meanwhile, it was already known that erythrocytes of most sheep and humans, unlike analogous cells of horses, guinea pigs, mice, and rabbits are very resistant to *Salmonella* cytolysin and therefore cannot be used for testing of this molecular ecological agent[16,17]. The authors focused on two closely related subjects: the optimal conditions for revealing the *Salmonella* cytolysin, and the species and individual diversity in cell sensitivity to destruction by this molecular ecological agent. The chief aim of their work was the action of the *Salmonella* cytolysin on cells of humans and a wide range of animal species. The rationale for comparing species and individuals was determined by the well-known existence of relevant differences in susceptibility to natural and experimental *Salmonella* infection. While the final molecular interface of "*Salmonella*-victim" ecology is located on the level of the cells of mesenchymal origin, erythrocytes appeared to be the most convenient experimental model, especially for a wide-range population study of microbe-cell interaction[16,18,19].

## EFFECT OF *SALMONELLA* CYTOLYSIN ON THE CELLS OF DIFFERENT SPECIES

Clinical isolates of five *S. enterica* belonging to serovars Paratyphy A (strain 50503), Paratyphi B (strain 42), Paratyphi C (strain 1212), Typhi D (strain 4446), and E (strain 337), as classified by Kauffmann[20], were obtained[16] from the State Institute of Standardization and Control of Medical Biological Preparation (Moscow, Russia).

The assays were carried out by incubating the *Salmonella* serotypes A, B, C, D, and E in the broth agar with the red blood cells inside the plates. The red blood cells of 33 guinea pigs, 47 rabbits, 1,059 hens, 600 mice, 2 cats, 7 dogs, 16 sheep, 22 horses, 2 monkeys, Macacus rhesus, and 1,301 humans, including 150 twins and 264 individuals who either had abdominal typhoid or other salmonelloses, were tested for susceptibility to destruction by cytolysins of the *Salmonella* serotypes A, B, C, D, and E. Cytolytic activity was scored by a clearance zone on erythrocyte agar plates.

The erythrocytes of these ten biological species proved to be very different in their specific or individual sensitivity to this kind of cytolysis[16,17].

All the representatives of the herbivorous group of species (guinea pigs, horses, rabbits, and white mice) had erythrocytes with pronounced sensitivity to lysis by *Salmonella*. In this group, the changes indu[illegible] by the *Salmonella* in the erythrocyte agar plates were characterized by over 3-mm zone of complete hemolysis around the bacterial growth. No individuals with resistant erythrocytes were revealed in this group. All the strains of *Salmonella* studied were able to destroy erythrocytes in the above four species[16,17]. The results demonstrated the stable ability of all five *Salmonella* strains to produce diffusable (i.e., extrabacterial) cytolysin. This statement is also in contradiction to the claims made by the researchers quoted above[9,10,11,12], who failed to detect the cytolytic capacity of *Salmonella* on a regular basis. Thus the assertion that *Salmonella* is not hemolytic in the blood agar[13,14] appeared to be incorrect.

In contrast, the erythrocytes of mainly omnivorous species (dogs, cats, hens, monkeys, and sheep) revealed individually different relationships to lysis by *Salmonella*. In this group, the changes induced by the *Salmonella* in the erythrocyte agar plates followed individually different patterns: (1) no change, no hemolysis; (2) weak hemolysis (zone of complete hemolysis beneath the bacterial growth); (3) 2- to 3-mm zone of complete hemolysis around the bacterial growth; (4) over 3-mm zone of complete hemolysis around the bacterial growth[16]. In most cases, erythrocytes revealed absolute immunity to this cytodestructive factor. Over 98% of the hens had absolutely resistant erythrocytes and only 1.6% revealed a weak sensitivity to lysis by some of *Salmonella* strains. The erythrocytes of the 16 sheep tested were absolutely immune to the destructive action of all five *Salmonella* strains tested, and only two sheep had sensitive erythrocytes: one sheep was sensitive to two strains, but the other one was destroyed by all five strains tested. This observation allows for an explanation of the “irregularity” of *Salmonella* cytolytic capacity in the agar plates containing sheep blood.

Only some of individuals in this group of species had erythrocytes sensitive to the cytolysins of one or more of the *Salmonella* strains tested, and the sizes of the cytolysis zones varied from weak to pronounced[16]. This part of the discussed investigation also allows the supposition that cytolytic molecules produced by different *Salmonella* strains have differing potential for destroying the erythrocytes of the same individual, and, consequently, of different molecular structures. The results also demonstrated that all five *Salmonella* strains possess cytolytic activity, which can be restricted by species and individual features of erythrocytes.

The analogous individual variability of the sensitivity of human cells was revealed[16,17] in representatives in urban populations of Saint Petersburg (Russia), Kishinev (Moldova), Tartu (Estonia), and Alma-Ata (Kazakhstan). The erythrocytes of 1,037 healthy humans, including 887 healthy blood donors aged 25 to 40 years (Saint-Petersburg, Alma-Ata), pupils aged 14 to 17 years (Tartu, Kishinev), and 150 adult twins were tested. Nearly 90% of the 1,037 healthy individuals had erythrocytes resistant to lysis by one or more *Salmonella* strains tested. For instance, the incidence of sensitivity to cytolysin of *Salmonella* serovar D (*S. typhi*, the abdominal typhoid infecting agents) in the populations of Alma-Ata (Kazakhstan), Tartu (Estonia), Kishinev (Moldova), and Saint Petersburg (Russia) were 4.9, 13.7, 11.6, and 10,7%, respectively[16,17]. In association with the data on interspecies individual differences in the sensitivity of omnivorous animals, these observations also allow for the supposition that cytolytic molecules produced by different *Salmonella* strains have differing potential to destroy erythrocytes in the same individual and, consequently, have differing molecular structures. The results allow also the assertion that the victim’s target ecological molecule for *Salmonella* cytolysin is present on the mesenchymal cells of herbivorous but is either absent on the relevant cells of most representatives of omnivorous species or its molecular constitution does not match the ecological effector molecule from the infectious agent. This conclusion shows the way to discovery of the origin both of the target ecological molecule and its genotypic programmer.

The existence of those individual and interspecies differences may explain why *Salmonella* cytolysin cannot be regularly detected in human or sheep blood agar[9,10,11,12].

Unfortunately, the above data[17] had been presented without any molecular ecologic, pathogenetic, immunologic, and evolutionary interpretation and therefore they did not influence the development of knowledge about salmolysin and its role in molecular ecology of *Salmonella*-victim confrontation for a quote of century. Despite those interpretations published in some monographs[21,22], the existence of *Salmonella* cytolysin was only recognized just recently after the above differences between species were taken into account[15]. But the data[16,17] that had been neglected before can also be a base for some other important conclusions concerning genetic, ecologic, immunologic, pathogenetic, and evolutionary aspects of the *Salmonella*-victim ecological system.

## GENETICS OF IMMUNITY/SENSITIVITY TO *SALMONELLA* CYTOLYSYN

The earliest report on an inherent basis for genetic origin of sensitivity/immunity to *Salmonella* infection in animals was published over 70 years ago. Irwin[23] reported that rats that survived induced infections of *Salmonella* produced progeny more immune to the pathogen than the average population from which the parent had come, and proposed that the parents had been selected for inherent immunity. There is now a great deal of evidence that many humans and animals have specific properties of genetic immunity to infectious diseases[7,24,25,26,27]. Those inherent mechanisms for protection are of a constitutional nature. They develop during ontogeny, without the influence of a particular parasite or its metabolites, but according to genetic program of individual development[19,27,28,29]. They are present in the body before the attack of disease-causing agents. Individual differences, both in susceptibility to infectious diseases and in the severity of the clinical course, are predestined by those constitutional features[27,28,29,30,31]. Studies over almost 70 years have led to the identification of various genes that affect genetic immunity/sensitivity to *Salmonella* infection[31,32,33,34].

The above-discussed differences in sensitivity to cytolysin, which were revealed on an interspecies basis, allow for the hypothesis that there is a genetic origin for the differences between cells of different species and individuals. The recent report on an inherent basis for genetic immunity to *Salmonella* infection in animals contained information on a number of inbred lines of chickens that have been shown to be resistant or susceptible to systemic salmonellosis caused by *S. enterica* serovar Gallinarum in adult birds, or by *S. enterica* serovar Enteritidis and *S. enterica* serovar Typhimurium in young chickens. Immune lines show only a moderate pathology and low mortality rates, whereas susceptible lines display extensive pathological changes and higher levels of mortality following *Salmonella* infection. Genetic immunity to the salmonelloses in chickens was dominant and not linked to sex, MHC, or Slc11a1 (formerly known as Nramp1), which leads to resistance in mice and other species. A novel locus-encoding immunity to salmonellosis has been identified on chicken chromosome 5 and designated SAL1. The nature of the differences in pathology found between immune and susceptible chicken lines *in vivo* indicates that resistance is expressed at the level of mesenchymal cell — the mononuclear phagocyte system. Macrophages from adult resistant-line birds cleared *Salmonella* serovar Gallinarum from infected macrophages within 24 h, whereas *Salmonella* bacteria persisted within macrophages from susceptible-line birds for at least 48 h[31].

Up to 1978, there were no studies carried out on the genetic origin of population and individual diversity in human cell immunity/susceptibility to *Salmonella* cytolysins. The relevant characteristics discovered[16,17] and analyzed above did not show any essential correlation with blood group or factor, age, sex, or nationality of the persons examined, nor with the presence of anti-*Salmonella* agglutinins in their sera. The immunization of the Tartu group of subjects with typhoid-paratyphoid A and B vaccine did not change the interaction between erythrocytes and cytolysins; the test for erythrocyte sensitivity was repeated three times for the same persons before the vaccination and again 1 year afterwards, and stereotypically gave the same results[16,17]. With regard to species and individual diversity in the erythrocytes' immunity/sensitivity to destruction by *Salmonella* cytolysin and the characteristics of distribution among healthy and vaccinated persons, the data can confirm the genetic nature of individual factors, providing relationships between cells and *Salmonella* cytolytic activity.

The genotype dependence of resistance/sensitivity to *Salmonella* cytolysins was also confirmed by testing for susceptibility in the twins[16,17]. In a group of human twins, the incidence of individuals with sensitive erythrocytes correlated to that ascertained for other healthy persons. Out of 44 pairs of heterozygotic twins, persons with sensitive erythrocytes were found with the same frequency as other healthy people. The erythrocytes of 37 pairs of dizygotic twins (84.1%) were resistant. Sensitivity to one or more strains was found in 7 pairs (15.9 %), but none of these pairs showed concordance with this characteristic. For instance, in the first pair of dizygotic twins, the erythrocytes of one twin were lysed by *Salmonella* groups B, D, and E, while the other twin showed sensitivity only to *Salmonella* group E. In the fourth dizygotic pair, one twin was resistant to all *Salmonella* cytolysins tested, whereas the other twin showed sensitivity to cytolysins D and E. As for the group of 62 monozygotic twins, sensitivity was attributed to 4 pairs (12.9%). Here all 4 sensitive pairs had full double concordance for this characteristic. For instance, in the third and fourth twin pairs in this group, the erythrocytes of both twins were lysed only by *Salmonella* of types D and E, but were absolutely immune to cytolysins types A, B, and C. The results also demonstrated both the differences between salmolysins of different *Salmonella* types and the genetic origin of diversity in individual sensitivity to these molecular ecological agents of *Salmonella*.

## IMMUNOLOGIC, PATHOGENETIC, AND EVOLUTIONARY CONSEQUENCES

The possible pathogenetic role and epidemic significance of the differences between individuals was characterized through an examination of 264 people who had abdominal typhoid or other salmonelloses before the testing. These individuals demonstrated lysability of their erythrocytes on a more frequent basis than the other members of the sample population. For example, of the 149 Alma-Ata inhabitants who had abdominal typhoid, sensitive erythrocytes were revealed at a rate of 70.5%, which is 4.4 times more frequent than the same city inhabitants who have never had the disease in their anamnesis, a rate of 15.9%. Analogous data have been revealed by examination of 94 salmonelloses reconvalescent[16].

The high frequency of persons with sensitive erythrocytes among those who have previously had salmonelloses cannot be explained by the influence of infectious disease as a factor that could supposedly change initially resistant erythrocytes, but rather by the fact that people with *Salmonella*-sensitive erythrocytes have higher receptivity to *Salmonella* infection. The data can also substantiate the genetic origin of individual sensitivity/immunity of human erythrocytes to *Salmonella* cytolysins and their correlation with the intensity of the appropriate epidemic process.

Any infectious agent only causes disease if the both the effector ecological molecule from the agent and the victim's target ecological molecule match. If the victim does not have compatible ecological molecule, then the victim's molecular constitution will block the infection providing constitutional genetic immunity[21,27]. While salmonelloses affect their victims by means of nutrition (via the alimentary tract), the origin of differences in cell sensitivity to *Salmonella* cytolysin discovered between mainly herbivorous species (guinea pigs, horses, rabbits, and white mice) and mainly omnivorous ones (humans, dogs, cats, hens, monkeys, and sheep) can be considered as a result of natural selection performed by regular interaction in this specific "microbe-victim" ecological system. The above data also confirm both the genetic origin of diversity in species and individual sensitivity to this molecular ecological agent of *Salmonella* virulence and the leading ecological function, i.e., pathogenetic significance of salmolysin, that was only hypotesized by some investigators[8,9,10,11,12].

The totality of the above-considered data allows for the hypothesis that the genetically determined individual characteristics of erythrocyte sensitivity to *Salmonella* cytolysins can serve as a marker of the constitutional predisposition for contamination by the infectious agent. In addition, the detection of resistant erythrocytes can substantiate the genetic immunity to disease. These data suggest a genetic tie to the distinction in sensitivity of erythrocytes to *Salmonella* cytolysins and also a connection between this phenomenon and some constitutional features of organisms that ensures genetic immunity against the appropriate infectious agent. One could also suggest that the high frequency of resistance in human and other omnivorous populations can be attributed to the natural selection operating in every epidemic[16,17]. This puts into question the need for a more detailed study on the genetic origin and epidemic meaning of the differences in cell sensitivity of different organisms to the *Salmonella* cytolytic toxin.

Species and individual diversity in cell sensitivity to *Salmonella* cytolysin can help in understanding some intriguing pathogenetic, epidemiologic, and clinical observations. The data can serve to orient molecular biological research in the evolution, epidemiology, and pathogenesis of *Salmonella* infection. Testing for individual differences in resistance/sensitivity to *Salmonella* cytolysin[16,17] promotes an approach to compare the coding genome sequences of individuals with high cell sensitivity and sequences of individuals with opposite cell characteristics, thus revealing the molecular determinants of relevant cell quality and creating a way to decipher the molecular target of Salmonella cytolysin on the cell envelope.
